# The Summer Session

**Published:** 1895-04-13

**Authors:** 


### THE SUMMER SESSION.

The medical schools are already making arrangements for the opening of the summer session of 1895 on May 1st. At St. Thomas's Hospital, students entering in the summer are eligible to compete for the Science Scholarships of £150 and £60 awarded in October, and other scholarships and prizes, to the value of over £500, are offered for annual competition. At St. Bartholomew's Hospital, scholarships for chemistry, biology, physics, of varying values from £20 to £150, are announced. Every advantage in the way of lectures and practical laboratory classes is within the reach of the students. The London Hospital, "the largest in the kingdom," announces special classes for the University of London examinations, and twenty-six scholarships and prizes are given annually. Special arrangements are made to enable students entering in May to present themselves for examination in July. A reduction of 15 guineas is made to the sons of members of the profession. At the Middlesex and St. Mary's Hospitals similar advantages are offered to intending students. In the case of the first-mentioned school students from the Universities of Oxford and Cambridge entering in May are eligible to compete for the University Scholarship of £60 awarded at the commencement of the ensuing winter session.

